# A concept to transfer a therapeutic splint position into permanent occlusion with a customized lingual appliance

**DOI:** 10.1186/1746-160X-8-16

**Published:** 2012-05-21

**Authors:** Tina Sachse, Rainer Schwestka-Polly, Stefanie Flieger, Dirk Wiechmann

**Affiliations:** 1 Department of Orthodontics, Hannover Medical School, Hannover, Germany; 2 Department of Orthodontics, University of Münster, Münster, Germany

## Abstract

**Introduction:**

The role of occlusion concerning temporomandibular disorder is still unclear but seems to be the only component of the stomathognathic system dentists are able to change morphologically. The aim of the paper is to describe the orthodontist’s approach for transferring and maintaining a therapeutic splint position into permanent occlusion using a fully customized lingual appliance.

**Methods:**

Fixed acrylic bite planes on lower molars were used to maintain a symptom-free condyle position prior to orthodontic treatment. Silicone impressions of the arches including the fixed bite planes were used for the Incognito laboratory procedure. Two digital setups were made. One setup represents the target occlusion. A second setup including the bite planes was used to fabricate an additional set of lower molar brackets. In the leveling stage all teeth except the lower molars were settled to maintain the therapeutic condyle position. Finally, the fixed bite planes were stepwise removed and molar brackets were replaced to establish the permanent occlusion planned with the first setup.

**Results and discussion:**

The advantage of an individual lingual appliance consists in the high level of congruence between the fabricated setups and the final clinical result. Both the individual scope for design and the precision of the appliance were vitally important in the treatment of a patient with a functional disorder of the masticatory system.

## Introduction

Temporomandibular disorder (TMD) is a widespread and common disease and therefore also an economic problem for the health care system. The prevalence of pain associated with muscles of the temporomandibular region is about 10% and 11.4% of the population suffer from a disc displacement with reduction [1]. Women are more likely to suffer from TMD than men [2].

The clinical signs and symptoms of TMD are heterogenous. Most patients report of pain in the muscles of the stomathognathic system (i.e. myalgia) or pain or dysfunction of the temporomandibular joint (i.e. arthralgia, arthritis, disc displacement with/without reduction). Additionally other factors, like trauma, psychological or orthopedic disturbances can affect TMD [3].

The modern aetiological concept of TMD is mainly based on research by Dworkin and LeResche in the 1990s. Their research diagnostic criteria for temporomandibular disorders (RDC/TMD) considers somatic as well as psychological and social co-factors. The first axis includes a clinical examination that focuses on the muscles of the temporomandibular region and the status of the temporo-mandibular joint (pain/dysfunction). The second axis mainly focuses on the patient’s history including a graded chronic pain status, a jaw disability checklist, tools to evaluate the patient’s depression and non-specific physical symptoms and a checklist to gain detailed demographic information [4].

Today the RDC/TMD are commonly used by clinicians and investigators over the world since there are many translations available such as the German version [5].

Although occlusion remains unconsidered in the RDC/TMD instrument, dentists traditionally focus on it as a major aetiological factor. In addition, occlusion seems to be the only component of the stomathognathic system they are able to change structurally and morphologically.

As the role of occlusion is still unclear [6], the clinician should be careful in changing the patient’s occlusion irreversibly from the beginning. A temporary and reversible improvement towards a well-balanced static and dynamic occlusion should be the first step, which is mostly realized by a customized acrylic splint fabricated after registration of the centric condylar position (CCP). The patient is instructed to wear the splint several hours in the daytime and the whole night. In many cases this treatment leads to a relief of pain and an improvement in joint function [7].

This first therapeutic step might then be followed by other, more invasive and irreversible procedures such as prothodontics or orthodontics. These therapeutic options apply especially to patients who require prosthodontic or orthodontic intervention a priori. The sticking point of these interventions following splint therapy is precisely transferring and therefore maintaining the CCP.

The following article describes the orthodontist’s approach for transferring and maintaining a therapeutic condyle position into permanent occlusion using the Incognito-System (3M Unitek, Monrovia, USA).

## Materials and methods

### Patient

A 24-year-old female patient was referred to the Hannover Medical School for orthodontic treatment after successful splint therapy. Her symptoms prior to treatment comprised pain during mastication in the left TMJ. A painful capsulitis with a backward and upward condyle displacement in maximal intercuspidation was diagnosed. The patient’s individual loading vector was determined dorso-cranially, and ventro-caudal positioning of the mandible was achieved by means of a removable positioning splint.

**Figure 1:**
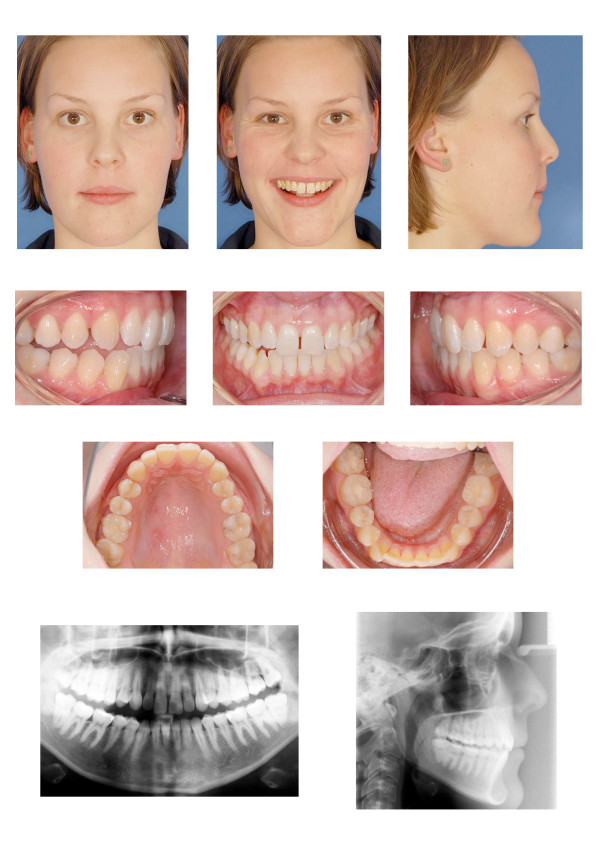
**The occlusal relationship in the new therapeutic condyle position shows a slight bilateral class III molar relationship, an open bite on the right side with an edge to edge transverse discrepancy, a lower incisor midline shift to the right side, lower incisor crowding and a diastema.** The cephalometric analysis reveals a slight maxillary protrusion.

The acrylic splint was worn full time and the symptoms were remediated. The occlusal relationship in the new therapeutic condyle position was as follows: Slight bilateral class III molar relationship, open bite on the right side with an edge to edge transverse discrepancy, lower incisor midline shift to the right side, lower incisor crowding and a diastema (Figure 1).

### Treatment objective

The first step of the treatment plan was to maintain the new therapeutic position with fixed acrylic bite planes on the lower molars. If the patient remains symptom-free an orthodontic treatment is possible. The occlusion in the new therapeutic condyle position could be seen as the initial malocclusion for the following fixed appliance treatment.

The aim of orthodontic treatment in this case is to stabilize the therapeutic position of the mandible - and consequently both joints – by establishing a defined occlusal relation. Treatment is conducted using the fully customized lingual appliance as described by Wiechmann [8,9]. This appliance will serve to transfer the splint-borne position to a defined occlusal relation.

### Fabrication and fitting of the fixed acrylic splints

Acrylic bite planes for the lower first and second molars are fabricated on plaster models mounted in a semi-adjustable articulator using a face bow and centric registration.

**Figure 2:**
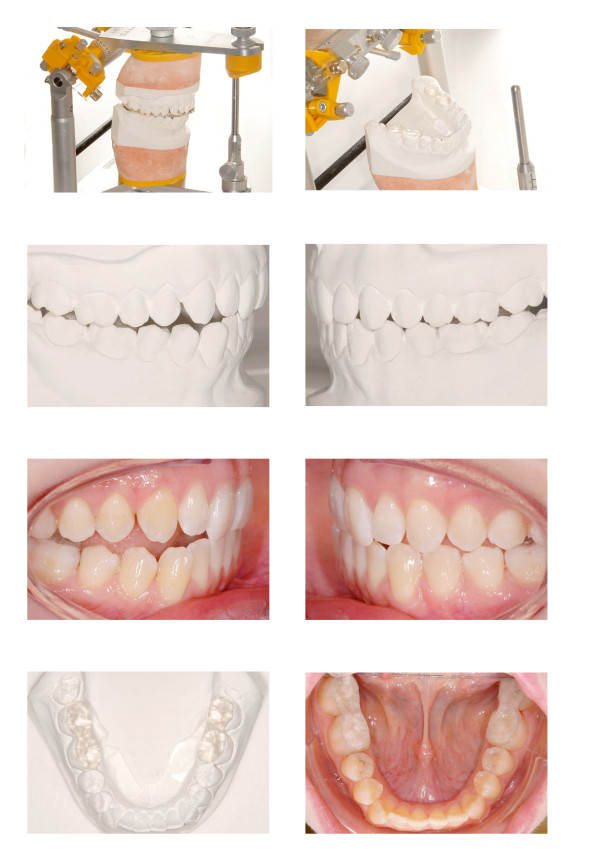
Acrylic bite planes for the lower first and second molars are fabricated on plaster models mounted in a semi-adjustable articulator using a face bow and centric registration.

The plaster casts were insulated prior to the fabrication of the splint surfaces using the “AislarR” alginate separating liquid (Fa. Hereaus-Kulzer GmbH, Hanau, Deutschland). The transparent powder and liquid component of “ForestacrylR” (Fa. Forestadent, Pforzheim, Deutschland) served to construct the acrylic surfaces which were adapted to the crowns of the lower first and second molars (Figure 2).

The occlusal surfaces of the lower first and second molars were cleaned and sand blasted using aluminium oxide particles of 50 μm diameter. The DRY-Field-System (NOLA, Greatlakes Orthodontics, Ltd, Tonawanda, New York, USA) is inserted, and the occlusal surfaces are etched by applying 37% phosphoric acid gel for 20 seconds. The acid gel is then rinsed off thoroughly and the occlusal surfaces are dried. The adhesion surfaces of the splints are sand blasted and coated with plastic conditioner. Light or dual curing resin, e.g. Nexus NX3 dual cure (Kerr, Inc, Orange, CA, USA), serves to attach the acrylic material to the tooth surfaces (Figure 2).

### Maintaining the therapeutic tmj position with the fully customized lingual appliance

After sonic scaling and air flow cleaning of the teeth, addition cure silicone impressions are made. These provide the foundation of the subsequent dental laboratory procedure.

In addition, a mandibular plaster cast representing the situation before insertion of the bite planes is required. For this purpose, an alginate impression provides a sufficient level of accuracy, and an appropriate plaster cast can already be fabricated in the course of the construction of the bite planes.

Maxillary and mandibular corrective copings as well as the mandibular plaster cast and a laboratory form are sent to 3M - Top Service GmbH, Bad Essen for further processing.

**Figure 3:**
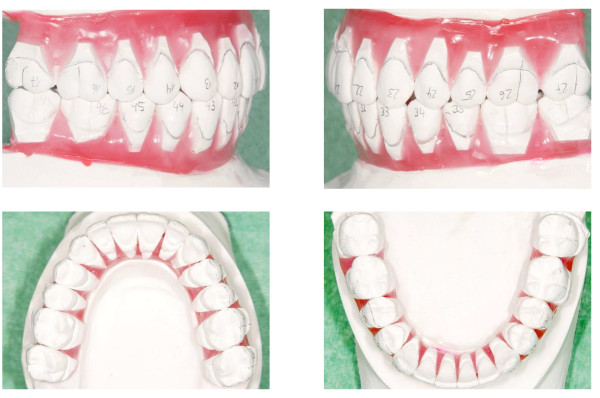
The target setup represents the final occlusion in the therapeutic condyle position.

**Figure 4:**
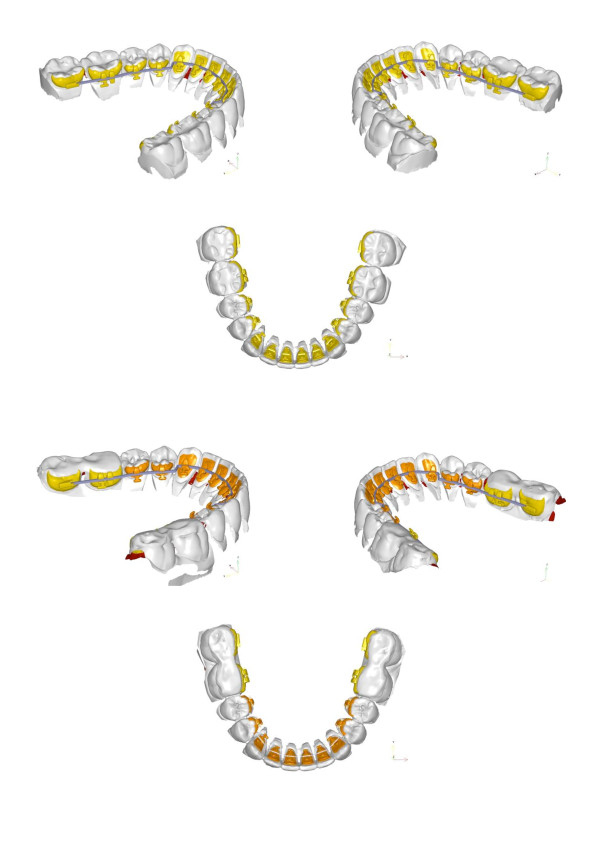
**The two digital setups with digital brackets placed.** The upper row represents the target setup. The lower row represents the setup with the acrylic bite planes on the lower molars.

There, therapeutic setups are fabricated (Figure 3) and scanned, the first of which represents the aspired final situation. For the second setup, the molars without bite planes are replaced with those having bite planes (Figure 4).

**Figure 5:**
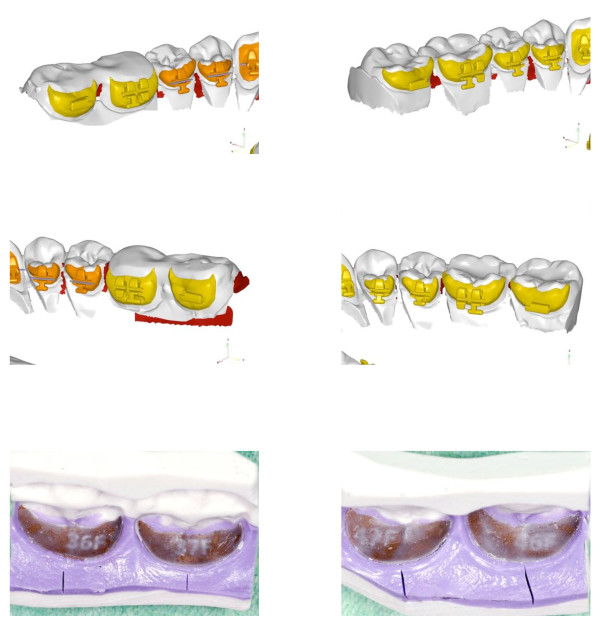
**Details of the different bracket positions.** Lower molars with acrylic bite planes (left column) and without bite planes (right side). The slot of the lower molar brackets fitted with bite planes were positioned further coronally than in the brackets produced for the final situation.

The second setup also permits for fabrication of an additional set of lower molar brackets fitted with bite planes and a slot which is positioned further coronally than in the brackets produced for the final situation (Figure 5).

### Orthodontic treatment with the a fully customized lingual appliance

**Figure 6:**
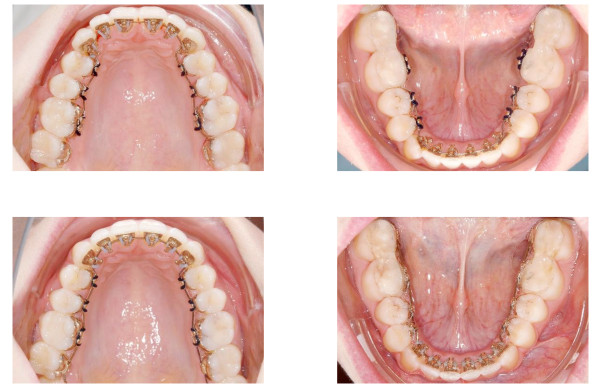
**Upper row: The first of the individually fabricated archwires are .014" SeNiTi wires.** They are placed in the lower incisor and canine brackets’ self ligating slot. Lower row: Later, treatment is continued with .016"×.022" SeNiTi wires.

Orthodontic treatment is commenced by inserting the bite plane fitted set of lower molar brackets and the remaining mandibular brackets. After four weeks, treatment is continued by inserting the maxillary brackets. The first of the individually fabricated archwires are .014" SeNiTi wires (Figure 6). They are placed in the lower incisor and canine brackets’ self ligating slot. The maxillary arch wire is inserted usind tip-top-tie ligatures in the frontal segment. The tip-top-tie ligature consists of four power chain (Rocky Mountain Orthodontics, Inc, Denver, Colorado, USA or Ormco, Inc, Orange, California, USA) modules, which are attached to the hook and to the occlusal wing of the bracket just like in the german overtie but with the archwire placed behind the occlusal wing rather than in the slot. The ligature is pulled from behind the wing, around the archwire and down to the hook, locking the wire behind the wing. The three remaining elastic modules are removed. The tip-top-tie is indicated in cases of pronounced anterior crowding or when an expansion of the dental arch is desired. Both wires are replaced into the main slot on a subsequent appointment. Later, treatment is continued with .016"x.022" SeNiTi wires (Figure 6).

**Figure 7:**
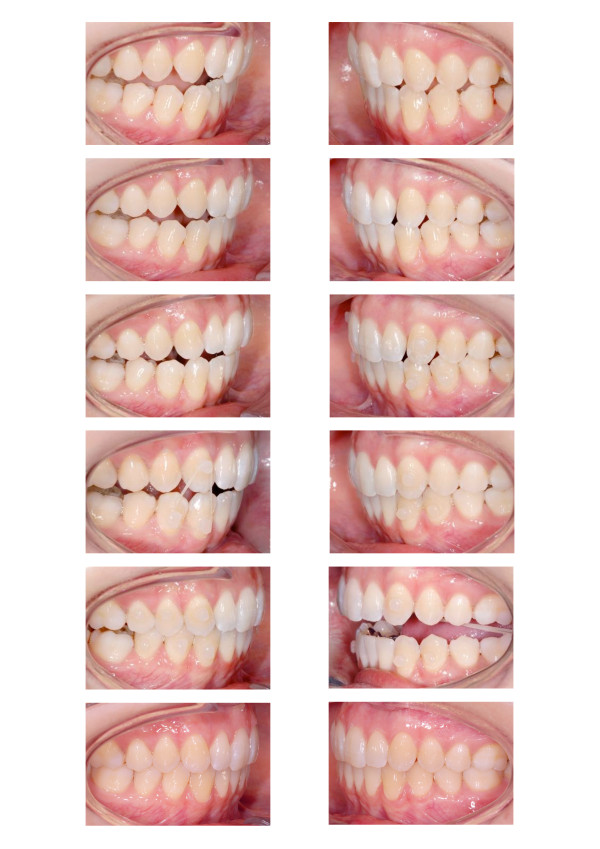
**Overview of the treatment progress.** Intermaxillary elastics are placed on buccally bonded resin attachments. Once occlusal support is established at the bicuspids the bite planes on the lower second molars are removed.

**Figure 8:**
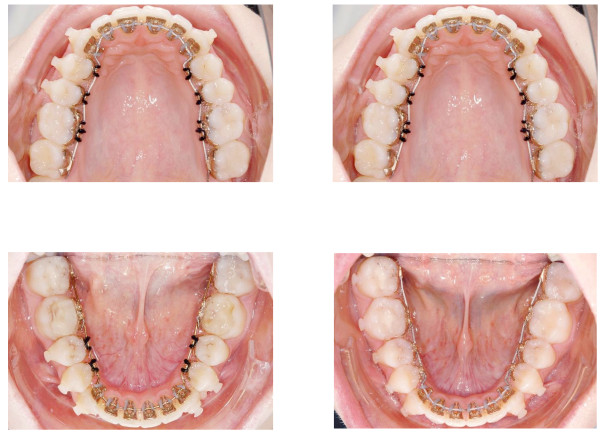
**Once occlusal support is established at the bicuspids (left column) the bite planes on the lower second molars are removed and the brackets replaced (right column).** Finishing and torque control are performed by inserting a slot filling .0182"x.0182" TMA wire.

The main task of the leveling stage consists in the stabilization of the vertical dimension at the bicuspids determined by the bite planes and settle maxillary and mandibular teeth in a fashion that permits for removal of the acrylic material while maintaining to the therapeutic occlusal relation. This process is enforced by intermaxillary elastics (1/8″, 6 oz, FA. Forestadent, Pforzheim, Germany). These are placed on buccally bonded resin attachments (Mini-Mold Buttons-Kit, G&H Wire Company, Franklin, Indiana, USA). The treatment progress is shown in Figure 7. Once occlusal support is established at the bicuspids the bite planes on the lower second molars are removed and the brackets replaced. A replacement of the archwire is not necessary at this time. Once the second molars are brought into the desired occlusion the lower first molars undergo bite plane removal and bracket change. At this stage, the therapeutic occlusion has been transferred to occlusal relation. Finishing and torque control are performed by inserting a slot filling .0182"x.0182" TMA wire (Figure 8).

**Figure 9:**
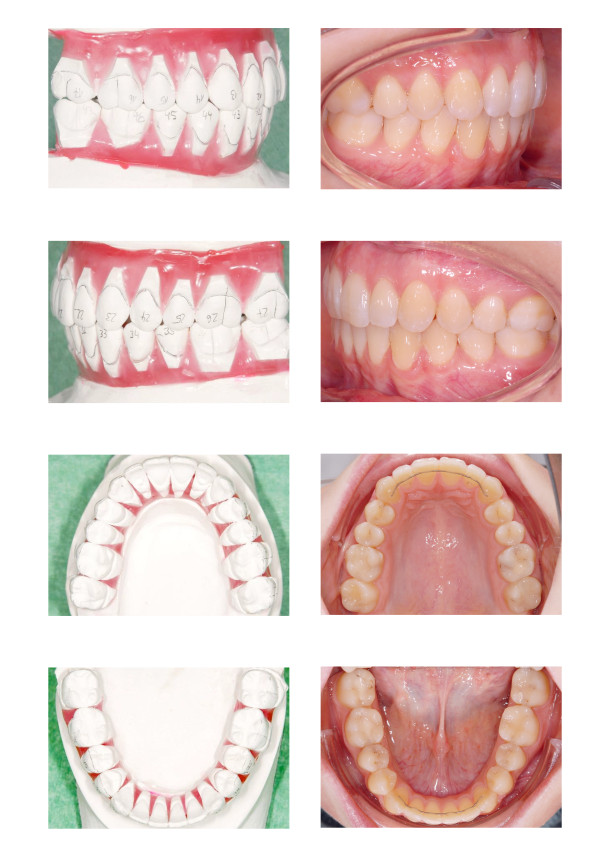
Comparison of the setup and the post-treatment occlusion.

**Figure 10:**
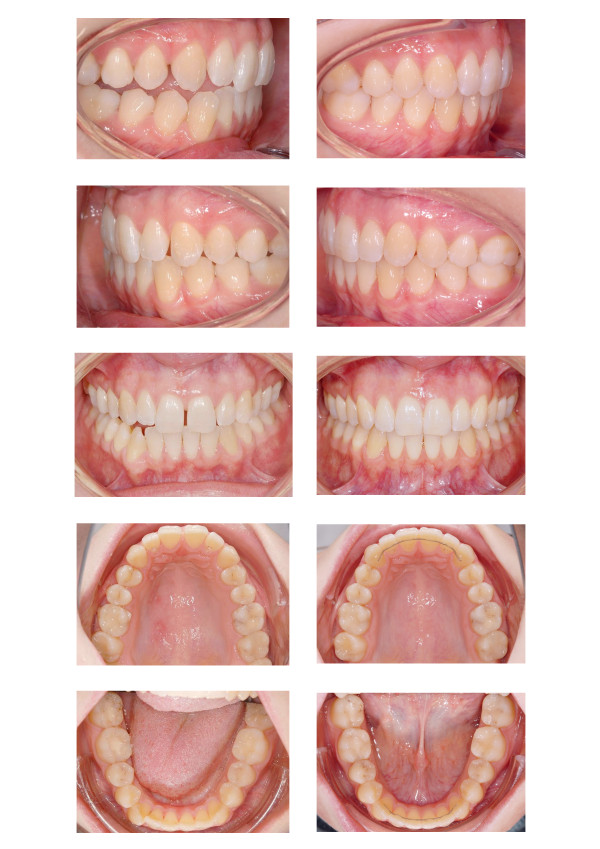
Comparison of the pre- (left) and the post-treatment occlusion (right).

**Figure 11:**
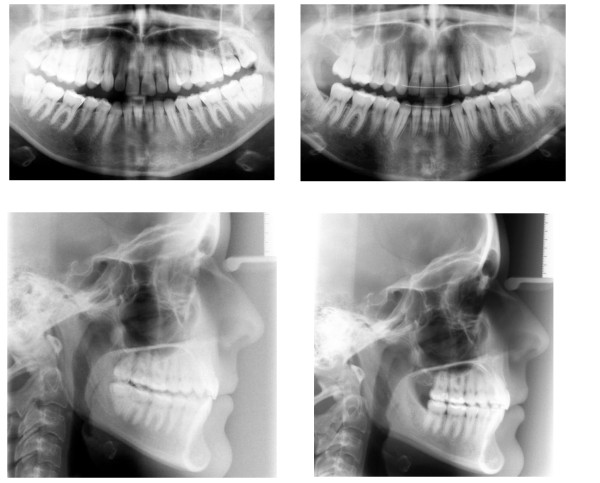
Comparison of the pre- (left) and the post-treatment xrays (right).

Long time retention is achieved through bonded maxillary and mandibular 6 point retainers. To maintain sagittal and vertical relations the patient is provided an activator for nightly wearing. A comparison of the setup and the post-treatment situation is shown in Figure 9. Figures 10 and 11 display the pre- and post-treatment situations.

The treatment was chosen according to the patient’s needs. No part of the therapy was conducted solely for the sake of research. Therefore, no ethical approval was sought.

## Consent

Written informed consent was obtained from the patient for publication of this report and any accompanying images.

## Discussion

In orthodontics, fixed and removable appliances are used, and both of these groups feature advantages and shortcomings. Equally, removable splints and fixed bite planes exhibit different properties, some of which constitute advantages in favor of fixed treatment.

Fixed bite planes are permanently effective as they do not need to rely on patient compliance. They also permit for a more accurate adjustment of the vertical dimension, thus providing the means for an ideal preparation of the intended reconstruction. However, being fixed, they are more difficult to modify or repair. Patients wearing fixed bite planes are required to take regular appointments. Three months of treatment time may be enough to establish irreversible changes in tooth positions such as intrusion or extrusion.

The shift from a removable splint to fixed bite planes did not entail the re-occurrence of symptoms. The patient remained pain free during the following two year phase of orthodontic treatment. The post-treatment functional status provided no evidence of any remaining structural lesions, loading vectors or restriction vectors.

The advantage of an individual lingual appliance consists in the high level of congruence between the initially fabricated setup and the final clinical result [10]. Both the individual scope for design and the precision of the appliance were vitally important in the treatment of a patient with a functional disorder of the masticatory system. After the removable splint had been replaced with fixed bite planes it was necessary to maintain the mandibular position and, consequently, the centric condyle position during the second phase of treatment. The fabrication of different set ups as well as the preparation of two different sets of brackets for the lower molars with slots at different levels permitted for a purposeful movement of the remaining teeth.

## Conclusions

The transfer of a therapeutic splint position to centric occlusion may be performed accurately by means of the individual lingual fully customized lingual appliance. The presented therapeutic options and results indicate the Incognito-Appliance to be a useful therapeutic option.

## Competing interests

The authors declare that they have no competing interests.

## Authors’ contributions

DW and RSP suggested the original idea for the paper. TS and DW treated the patient. TS made all medical records and wrote the main part of the manuscript. RSP and DW reviewed and contributed to the writing of all iterations of the paper. SF wrote parts of the paper, made the literature search, contributed to the English translation, reviewed the paper for content, including the final version of the manuscript. All authors read and approved the final manuscript.
